# THE DYSPHAGIA INVESTIGATION: IS THERE STILL SPACE FOR THE VIDEOFLUOROSCOPIC METHOD?

**DOI:** 10.1590/0102-672020210002e1650

**Published:** 2022-06-17

**Authors:** Charles Henrique Dias MARQUES, Luiz João ABRAHÃO-JÚNIOR, Eponina Maria Oliveira LEMME

**Affiliations:** 1 Federal University of Rio de Janeiro, Digestive Motility Laboratory - Gastroenterology Division of HUCFF-UFRJ, Rio de Janeiro, RJ, Brazil

**Keywords:** Deglutition Disorders, Evaluation Studies as Topic, Gastroenterology, Transtornos de Deglutição, Estudos de Avaliação como Assunto, Gastroenterologia

## Abstract

Dysphagia can lead to aspiration pneumonia, impacting the nutritional status and quality of life of the patient. The videofluoroscopy is highlighted for allowing both a real-time evaluation and the recording of the images for later review and analysis.

**AIM:**

This study aimed to describe the characteristics of the population referred for videofluoroscopy and its value as an investigation method.

**METHODS:**

A descriptive and retrospective study was conducted. Exams were analyzed in lateral and anteroposterior views and reviewed using the frame-by-frame analysis software. The variables analyzed were an indication of the exam, previous diseases, dynamics of the oral and pharyngeal phases, and the degree of penetration/aspiration.

**RESULTS:**

A total of 141 exams were analyzed. The study population had a median age of 66.24±17.78 years. For the indication of the exam, the investigation of dysphagia was highlighted (n=87, 61.7%) and for previous conditions, diverticulum (n=13, 9.2%), pharyngeal bar (n=12, 8.51%), and stroke and Parkinson’s disease (n=9, 6.4%) were highlighted. In the oral phase, 45 (31.9%) patients had a premature loss, and 108 (76.6%) patients had normal transit time. However, 100 (70.9%) had inadequate ejection. In the pharyngeal phase, 119 (84.4%) had efficient laryngeal displacement and 107 (75.9%) had an adequate opening of the upper esophageal sphincter. The beginning of the pharyngeal phase was classified as inadequate in 131 (92.9%) patients, and 80 (56.74%) had pharyngeal residue. Notably, 100 (70.9%) patients had grade 1 on the penetration/aspiration scale.

**CONCLUSION:**

Despite the didactic division of phases, swallowing is complex and has transition stages. Videofluoroscopy is the only method for evaluating all phases of swallowing and its events.

## RESUMO - RACIONAL

a disfagia pode levar ao desenvolvimento de pneumonia broncoaspirativa, impactando o estado nutricional e a qualidade de vida do paciente. A videofluoroscopia se destaca por permitir tanto a avaliação em tempo real como o registro das imagens para revisão e análise posterior.

**OBJETIVO:**

Descrever as características da população encaminhada para videofluoroscopia e seu valor como método de investigação.

**MÉTODOS:**

estudo descritivo e retrospectivo. Exames analisados em visão lateral e ântero-posterior e revisados com *software* de análise quadro a quadro. Variáveis analisadas: indicação do exame, doença base, dinâmica da fase oral e da fase faríngea e a grau de penetração/aspiração.

**RESULTADOS:**

foram analisados 141 exames. A população estudada tinha em média 66,24 ± 17,78 anos. Para indicação do exame, destacou-se: investigação de disfagia (n=87, 61,7%) e para condição prévia: divertículo (n=13, 9,2%), barra faríngea (n=12, 8,51%), acidente vascular e doença de Parkinson (n=9, 6,4%). Na fase oral, 45 pacientes (31,9%) apresentaram escape prematuro. Tempo de trânsito normal foi registrado em 108 (76,6%) pacientes. Porém, 100 pacientes (70,9%) apresentaram ejeção inadequada. Na fase faríngea, 119 (84,4%) apresentaram deslocamento laríngeo eficiente e 107 (75,9%) abertura adequada do esfíncter esofágico superior. O início da fase faríngea foi classificado como inadequado em 131 pacientes (92,9%) e 80 (56.74%) apresentaram resíduo faríngeo. 100 pacientes (70,9%) apresentaram grau 1 na escala de penetração/aspiração

**CONCLUSÃO:**

apesar da divisão didática das fases, a deglutição é uma função complexa e possui etapas de transição. A videofluoroscopia é o único método para avaliar todas as fases da deglutição e seus eventos.

## INTRODUCTION

In the past years, other methods for swallowing assessment have emerged, such as fiberoptic endoscopic evaluation of swallowing and high-resolution manometry; however, the experience with these new methodologies still demonstrates certain limitations, such as not allowing evaluation of all phases of swallowing, which interferes, in many cases, with the pathophysiological understanding and therapeutic approach^22^. In this sense, videofluoroscopic swallowing study (VFSS) is still presented as the main method for evaluating oropharyngeal dysphagia.

Swallowing is the process by which bolus is transferred from the mouth to the stomach. The swallowing process keeps a functional relationship between the digestive and respiratory systems. This aspect reinforces the main concern, in the specialized literature, about the pulmonary and nutritional health of this population^9^ ^,^ ^26^. In addition, dysphagia can create impacts on the patient’s quality of life and social aspects of the feeding process, resulting in isolation and depression^12^.

Dysphagia occurs when there is any abnormality in the swallowing mechanisms, which can be primary or secondary to an underlying disease. Difficulty in transporting food can occur in any of its phases, and because it is a dynamic process, the commitment in one of its phases impacts the others, which can result in damage to the entire process. It is noteworthy that dysphagia is often associated with higher mortality rates and prognostic limitation in several cases^23^.

VFSS is considered the gold standard method in the assessment of dysphagia^5^ ^,^ ^7^. Its main advantage is the real-time assessment of all phases of swallowing and the interface events between them, such as oral ejection and opening of the upper esophageal sphincter (UES). Although some authors report the use of radiation as a disadvantage, the videofluoroscopic method allows the recording of images for later review and analysis, avoiding new radiation exposure for patients and healthcare professionals^7^. The VFSS enables qualitative and quantitative analysis of dynamic swallowing events, including the identification of structural changes and the adaptation of rehabilitation strategies during the examination itself, and identifies penetration and/or aspiration.

The study of the swallowing function and its disorders has got greater attention in recent years, which is highlighted the attempt to establish a broader and consequently efficient approach. Therefore, assessments such as VFSS should be implemented at clinical centers that receive patients with dysphagia in their routine^23^.

This study aimed to describe the characteristics of the population that underwent this evaluation in a university hospital and the value of the videofluoroscopic method in the assessment of patients with dysphagia.

## METHODS

This is a retrospective and descriptive study conducted in the Esophageal Motility Laboratory of Gastroenterology Division at the Clementino Fraga Filho University Hospital of the Federal University of Rio de Janeiro. The study was approved by the Ethics Committee of the Institution (no 09780813.6.0000.5257). All medical records and contrast examinations sent for the evaluation of oropharyngeal swallowing were recovered, and for this study, only the VFSS exams were selected.

### Videofluoroscopic swallowing study

To perform the exam, swallows were evaluated with contrast (100% barium sulfate) in lateral and anteroposterior views. Images were recorded and captured in Audio Video Interleave format. This allowed reviewing and evaluating at a later time using the frame-by-frame graphic software, ensuring precision in the evaluation.

### Variables studied

The variables analyzed were an indication for the exam, disease diagnosis, dynamics and changes in the oral phase, dynamics and changes in the pharyngeal phase, and classification of the degree of penetration/aspiration.

The oral phase was considered presence or absence of premature loss. Types of oral organization are closed (bolus positioned directly on the back of the tongue); anterior opened (presence of anterior space, which is filled by contrast); anterior-superior (contrast in the space in front and above the tongue); elongated (contrast occupies the anterior region and extends to the soft palate); and unstable (contrast oscillates and there is no constancy in the oral organization). For oral transit time, it was considered adequate when the events of the oral phase occurred synchronously and inadequate in cases of abnormalities. Oral ejection was classified as adequate, slowed (when the contrasted bolus was transferred with reduced force and prolonged time), and multiple (when the content was transferred to the pharynx in more than one swallow). For the presence of oral stasis, it was considered after the complete transference of the bolus, the visualization of contrast in the oral vestibule, and tongue region.

For the pharyngeal phase, the laryngeal incursion was classified as adequate (when the hyolaryngeal complex presented competent incursion in terms of protective function) and inadequate (when it did not demonstrate competent incursion to protect the airways). For the UES opening, it was classified as adequate (when the degree of opening presented good dynamics in the opening of the anterior and posterior walls) and inadequate (when the UES did not present enough opening for the passage of the bolus). For the onset of the pharyngeal phase, it was considered adequate (when it started at the posterior angle of the mandible) and inadequate (when started on the valleculae, aryepiglottic folds or pyriform sinuses). The presence of pharyngeal residue was divided into absent or present (when there was a presence of contrast in the region of valleculae, aryepiglottic folds, and pyriform sinuses). The pharyngeal dynamics was considered adequate (when performed through both lateral walls) and inadequate (when the bolus passed only by one side, showing a unilateral contraction).

The Rosenbek’s Penetration and Aspiration Scale (PAS) was used to classify the degree of penetration and aspiration when observed. The PAS varies with grade 1 (contrast does not enter the airway), grade 2 (presence of contrast above the vocal folds without residue), grade 3 (contrast remains above the vocal folds with visible residue), grade 4 (contrast contacts vocal folds without residue), grade 5 (contrast contacting vocal folds with visible residue), grade 6 (contrast passes by the glottis but there is no residue at the subglottic level), grade 7 (contrast passes by the glottis with residue at the subglottic level and the patient presenting defense response), and grade 8 (contrast passes below vocal folds with residue in the subglottis, but the patient does not show a response).

### Statistical analysis

The statistical analysis applied was descriptive through the use of the absolute number for each variable studied and their respective percentages. To describe the characteristics of the studied group, mean and standard deviation were applied.

## RESULTS

A total of 292 exams were performed in the laboratory, from 2012 to 2018, of which 141 (48.3%) corresponded to the evaluation of swallowing using the VFSS (Table 1).

**Table 1 -:** Types of exams to assess swallowing.

Type of exam	N (%)
Videofluoroscopic swallow study	141 (48.3)
Timed barium esophagogram	87 (29.8)
Videoesophagogram	34 (11.6)
High-resolution videomanometry	30 (10.3)
Total	292 (100)

The main indication for VFSS was for the investigation of dysphagia in 87 (61.7%) patients. However, in 27 (19.1%) patients, it was not possible to identify the indication for the exam (Table 2).

**Table 2 -:** Indication for performing VFSS.

Indication	N (%)
Dysphagia	87 (61.7)
Gastroesophageal reflux disease	6 (4.3)
Hiatus hernia	6 (4.3)
Others	15 (10.6)
Unidentified data	27 (19.1)
Total	141 (100)

Among the diagnosed diseases, the highlight was the presence of esophageal diverticulum (9.2%), pharyngeal bar (8.51%), and stroke and Parkinson’s disease (6.4%). The remaining patients were grouped under other conditions, such as Guillain-Barré, oculopharyngeal dystrophy, obstructive sleep apnea, brain injury, and Wilson’s disease (3.6%) (Table 3).

**Table 3 -:** Diseases diagnosed in patients undergoing VFSS.

Diagnoses	N (%)
Esophageal diverticulum	13 (9.2)
Pharyngeal bar	12 (8.5)
Parkinson’s disease	9 (6.4)
Stroke	9 (6.4)
Amyotrophic lateral sclerosis	7 (5)
Osteophyte	6 (4.3)
Achalasia	6 (4.3)
Neoplasm	5 (3.5)
Dementia	3 (2.1)
Tracheostomy	3 (2.1)
Multiple sclerosis	2 (1.4)
Chagas disease	2 (1.4)
Scleroderma	2 (1.4)
Others	5 (3.6)
Unidentified data	57 (40.4)
Total	141 (100)

**Figure 1 -:**
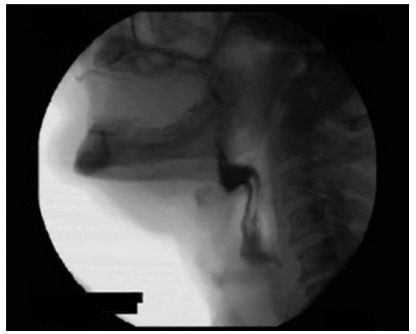
Premature loss of contrasted bolus.

For the evaluation of the oral phase, 45 (31.9%) patients presented premature loss of the ingested bolus (Figure 1). In 16 (11.4%), a closed type of oral organization was observed and other types were shown in 125 (88.6%). In 108 (76.6%) patients, oral transit was adequate. However, 100 (70.9%) patients had inadequate oral ejection and 34 (24.1%) had oral residue (Table 4).

**Table 4 -:** VFSS results for oral phase evaluation.

	Oral phase				
	Premature loss N (%)	Closed organization N (%)	Normal transit N (%)	Proper ejection N (%)	Oral residue/stasis N (%)
Yes	45 (31.9)	16 (11.4)	108 (76.6)	41 (29.1)	34 (24.1)
No	96 (68.1)	125 (88.6)	33 (23.4)	100 (70.9)	107 (75.9)

**Figure 2 -:**
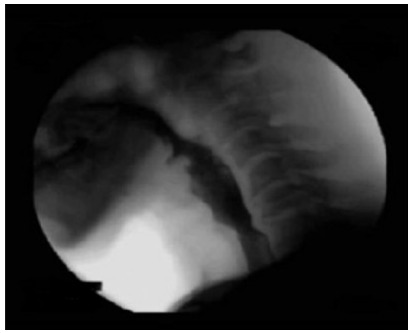
Upper esophageal sphincter hypertonia.

In the evaluation of the pharyngeal phase, 119 (84.4%) patients had efficient laryngeal displacement and 107 (75.9%) had an adequate opening of the UES, with 34 (24.1%) patients with incomplete or inadequate opening (Figure 2). The onset of the pharyngeal phase was mainly classified as inadequate in 131 (92.9%) patients. The pharyngeal residue was present in 80 (56.74%) patients, and the pharynx dynamics was adequate in 138 (97.87%) (Table 5).

**Table 5 -:** VFSS results for evaluation of the pharyngeal phase.

	Pharyngeal phase				
	Laryngeal incursion N (%)	UES opening N (%)	Onset N (%)	Pharyngeal residue/stasis N (%)	Pharyngeal dynamics N (%)
Adequate/yes	119 (84.4)	107 (75.9)	10 (7.1)	80 (56.74)	138 (97.87)
Inadequate/no	22 (15.6)	34 (24.1)	131 (92.9)	61 (43.26)	3 (2.13)

**Figure 3 -:**
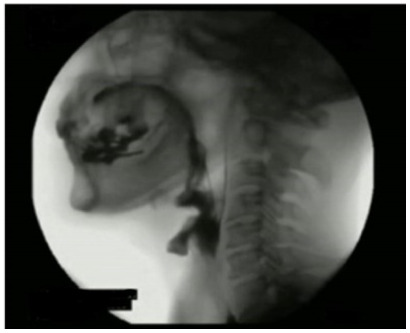
Penetration scale grade 5

**Figure 4 -:**
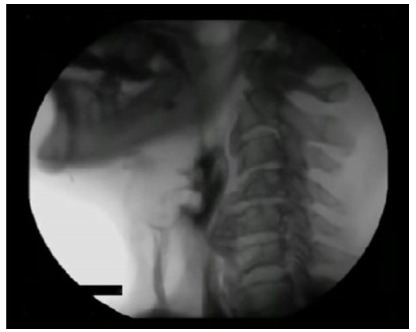
Aspiration scale grade 8

In the assessment of the degree of penetration/aspiration using the PAS score, the most frequent result was grade 1 in 100 (70.9%) patients. Nine (6.4%) had aspiration without the presence of residue at the subglottic level - grade 6. Two (1.42%) cases had grade 5 of penetration (Figure 3), and two other cases (1.42%) presented aspiration with no airway protection response - grade 8 (Table 6) (Figure 4).

**Table 6 -:** Results of the degree of penetration/aspiration (PAS).

Category	PAS Score	N (%)
Penetration	1	100 (70.9)
	2	21 (14.9)
	3	5 (3.55)
	4	0 (0)
	5	2 (1.42)
Aspiration	6	9 (6.4)
	7	2 (1.42)
	8	2 (1.42)

## DISCUSSION

This study analyzed 141 VFSS exams demonstrating its value in the evaluation of events related to the swallowing phases, as well as the description of alterations present in oropharyngeal dysphagia conditions. This aspect corroborates with other studies that highlight the importance of VFSS both as an assessment routine, as well as in the process of more efficient therapeutic management for patients with dysphagia^14^ ^,^ ^16^.

Although the literature highlights the importance and impact of dysphagia, in terms of both the patient’s clinical health and direct and indirect costs for the hospital care^21^, the objective assessment is not routinely available in all units that provide care to patients with dysphagia. Eisenhuber et al.^11^, in a study that aimed to assess the availability of VFSS to examine patients in Austria, identified that one of the aspects that influenced the availability of the exam was the size of the hospital, where VFSS was available mainly in large hospitals. These data are compatible with the reality of the environment in which our work was performed, but it is noteworthy that one of the challenges in the assessment of patients with dysphagia is the need to maximize the achievement of VFSS.

The casuistic of 141 patients included 55.32% (n=78) female patients, with a mean age of 66.24±17.78 years, where the main indication for performing the contrast test was dysphagia (61.7%) (Table 2). Eisenhuber et al.^11^ identified that the most frequent symptom was dysphagia (45% in Radiology Services). It is noteworthy that the indication of dysphagia to perform the VFSS is justified by the characteristic of the disorder and the predominant concern in the literature about laryngotracheal aspiration and its clinical impacts^9^ ^,^ ^30^. Other studies indicate that proper management reduces pulmonary complications and impacts the quality of life of this population^25^ ^,^ ^27^.

In addition, our studied casuistic had an average age above 65 years and a higher prevalence of females. These two factors can interfere with the swallowing process and justify some of our results. Previous research comparing asymptomatic men and women using VFSS suggested that women’ swallow liquids have a lower flow rate and smaller volumes when compared to men^8^. Fiorese et al.^13^ emphasize that, in addition to tooth loss and poorly adapted oral prostheses, physiological changes such as the increase in connective tissue and fat on the tongue can lead to impacts on mobility and strength of the tongue. Important changes can also occur in swallowing with aging, changes in chewing, lip sealing, laryngeal elevation, and loss of muscle mass; in these cases, it is more evident in the geniohyoid muscle and the tongue^19^, reinforcing our findings for changes in the oral phase of deglutition. Other studies also demonstrated the concern with the lung health of the elderly population related to cases of aspiration^10^ ^,^ ^20^.

Regarding the oral phase, our results highlighted the higher prevalence of non-closed types of oral organization. Yamada et al.^28^ report variations in organization and oral ejection of normal controls and patients. They describe that in the closed organization the bolus maintained its position without difficulty and was identified in 71.4% of swallows in healthy individuals. The authors concluded that this is the physiological pattern of individuals without dysphagia. In our study, 88.6% of the patients presented a non-closed pattern, which is justified by the characteristic of the sample, composed of patients with dysphagia.

The organization of the oral phase, in addition to interfering with the dynamics of the oral phase, can consequently impact the ejection and pharyngeal phase. In our study, 70.9% had inadequate ejection and 92.9% had an inadequate pharyngeal phase onset. These data can be justified because although there is a didactic division of the swallow phases, swallowing is a complex and continuous function with transition events between phases such as the oral ejection and opening of the UES. Yamada et al.^28^ also demonstrated that the pharyngeal phase begins with the increased pressure in the oropharynx determined by the oral ejection of food. Dantas et al.^8^ in a study with asymptomatic controls also demonstrated that the bolus volume interfered with the oral transit time and opening of the UES. Therefore, changes in oral ejection can be justified by relationships with other steps of the oral phase. The oral phase can be divided into capture, qualification, preparation, positioning, and ejection, in which an initial step such as uptake may interfere in the organization and consequently with the ejection. This aspect can justify the prevalence of premature loss of the bolus into the pharynx in the studied population (31.9%, n=45), since both the control and the driving force of the bolus take place at the oral cavity and, consequently, at the oral phase of swallowing. Our findings for the oral phase corroborate with a previous study on the impact of oral changes in the pharyngeal phase, which highlights that although the events of swallowing are integrated, it is observed that the oral phase has not been as valued as the pharyngeal phase^28^.

In the pharyngeal phase, we can highlight its sequential onset with a higher prevalence of inadequate pattern (92.9%, n=131) compared to adequate onset (7.1%, n=10). The data for inadequate onset were distributed starting on the base of the tongue (34%, n=48), valleculae (42.6%, n=60), aryepiglottic folds (7.8%, n=11), and pyriform sinuses (8.5%, n=12). It is noteworthy here that, within the inadequate pattern, the higher prevalence in the region of epiglottic valleculae can be justified by the characteristic of the population being over 65 years old, which is an aspect that is in agreement with the literature. The elderly population demonstrates significantly delayed swallowing response times^24^ and onset of the pharyngeal phase, for both asymptomatic and symptomatic patients, being more frequent in the valleculae^29^.

Although, from the physiology point of view and defense mechanisms, the level of aspiration risk is lower when associated with this region^18^, Han et al.^15^ reported that patients who survived a stroke with penetration and aspiration have a greater tendency to present stasis in the valleculae and pyriform sinuses. The authors suggest that it is necessary to study how the presence of residue in these regions can affect penetration and aspiration. These data can be reinforced by the higher prevalence of grade 1 (70.9%, n=100) in the assessment of PAS, demonstrated in the present study. A study by Benfield et al.^4^ on the profile of clinics that perform VFSS in the United Kingdom reported that half of the units use defined protocols, the majority of which are developed internally. However, the authors emphasize that the use of classification scales can help to improve analysis and management. According to the authors, scales were used frequently, especially the PAS. These data show agreement with our work, since in our routine we use the PAS, and we observed the presence of aspiration in 13 patients, with a distribution of grade 6 (6.4%, n=90), grade 7 (1,42%, n=2), and grade 8 (1.42%, n=2).

Data from previous studies correlate the presence of residue in pharyngeal recess to problems related to tongue strength in patients with Parkinson’s disease^1^ ^,^ ^2^. Argolo et al.^3^ described that among 69 patients with Parkinson’s disease 30.4% had absent/mild valleculae residue and 69.6% had moderate/severe valleculae residue. For pharyngeal recess, the distribution was 73.9% absent/mild and 26.1% moderate/severe. Our results showed that most patients presented pharyngeal residue (56.74%, n=80), with a more prevalent distribution in valleculae (41.25%, n=33), valleculae and associated pyriform sinuses (22.5%, n=18), and in valleculae, aryepiglottic folds, and associated pyriform sinuses (21.25%, n=17).

Abnormalities of laryngeal incursion (15.6%, n=22) and opening of the UES (24.1%, n=34), although similar compared to normal controls, deserve to be described because they represent an important defense mechanism of the airways for swallowing. Han et al.^15^, in a retrospective study with 58 stroke patients, reported the impact of impairment of the elevation of the larynx as a factor that increases the risk of penetration and aspiration. In the group with penetration and aspiration, 88% had reduced laryngeal displacement. Costa^6^ describes that there is an expressive amount of new morphological and functional concepts that can explain the mechanisms of swallowing. Concerning the UES, the suprahyoid with modulation of the infrahyoid muscles is responsible for the movement of the hyoid-larynx complex. This action sustains the UES opening depending on the type of bolus, volume, and viscosity and is also assisted by the pharyngeal muscles, the stylopharynx. Part of the justification for our findings can be related to the heterogeneity of our sample as well as the predominance of previous diseases that do not interfere more impactfully in these events. It is noteworthy that within the problems of opening the UES and, compared to the total sample, 12 (8.5%) patients had a cricopharyngeal bar. In our experience, the evaluation of this segment is part of our routine, but not all available protocols consider the UES in its description, as well as, they present a lot of variation in the standardization process.

A study on the experience of applying objective measures for swallowing found that the main changes were problems with pharyngeal constriction (34.5%) and deficits in the airway protection (65.5%). For more common conditions, reflux-related dysphagia (36%), nonspecific pharyngeal dysphagia (24%), and Parkinson’s disease (16%) have been described^17^. These data partially corroborate with our study, which described comorbidities of patients undergoing the VFSS exam and which can be justified by the characteristic of our service. In our practice, it is common, for example, the evaluation of patients who could be considered in a casuistic of referred esophageal dysphagia.

Although other methods have become widespread, there are limitations in evaluating all phases of swallowing and in identifying aspiration compared to VFSS. The presence of dysphagia is directly associated with higher rates of complications with more restrictive prognosis in terms of rehabilitation. Therefore, objective evaluation using VFSS still proves to be an important radiological method of clinical value and should be part of a routine in every service that has regular contact with patients with dysphagia.

## CONCLUSIONS

Our results indicated that the oral phase deserves to be highlighted in VFSS. The types of organization, in addition to interfering with the oral phase, can impact the pharyngeal phase. These results are justified because, despite the didactic division of the swallowing phases, it is complex and has transition stages. We emphasize that VFSS is the only method for evaluating all phases of swallowing and its events.
